# Siamese and triplet network-based pain expression in robotic avatars for care and nursing training

**DOI:** 10.3389/frobt.2024.1419584

**Published:** 2024-09-26

**Authors:** Miran Lee, Minjeong Lee, Suyeong Kim

**Affiliations:** ^1^ Department of Computer and Information Engineering, Daegu University, Gyeongsan, Republic of Korea; ^2^ Graduate School of IT Convergence Engineering, Daegu University, Gyeongsan, Republic of Korea

**Keywords:** human-robot interaction, care and nursing education, pain expression, robotic facial expression, SIAMESE network

## Abstract

Care and nursing training (CNT) refers to developing the ability to effectively respond to patient needs by investigating their requests and improving trainees’ care skills in a caring environment. Although conventional CNT programs have been conducted based on videos, books, and role-playing, the best approach is to practice on a real human. However, it is challenging to recruit patients for continuous training, and the patients may experience fatigue or boredom with iterative testing. As an alternative approach, a patient robot that reproduces various human diseases and provides feedback to trainees has been introduced. This study presents a patient robot that can express feelings of pain, similarly to a real human, in joint care education. The two primary objectives of the proposed patient robot-based care training system are (a) to infer the pain felt by the patient robot and intuitively provide the trainee with the patient’s pain state, and (b) to provide facial expression-based visual feedback of the patient robot for care training.

## 1 Introduction

Patient robots for care and nursing training (PRCNT) can be used for training and improving care abilities in interactions with patients, older adults, or care receivers, such as treatment, nursing, bathing, transferring, and rehabilitation. In terms of various care and nursing environments, an outstanding caregiver must have not only competent skills to provide adequate care and support but also ancillary qualifications such as reliability, stability, optimism, and communication with care recipients as follows:

• Reliability: Skilled caregivers must increase the reliability of their skills by empirically acquiring the required skills of treatment and care.
• Stability: Stable posture and facial expression can reassure the patient and create a comfortable environment when constantly communicating with the patient.
• Optimism: Caregiver with an optimistic disposition can positively change the depression or low moods and anxious psychology of a care recipient.
• Communication: Care recipients may experience pain or stress in care or nursing environments, and caregivers must interact with them based on communication.

To achieve these abilities and qualities, experts or students in care and nursing need to learn and train to reach their superior skills consistently. CNT is to develop the ability necessary to effectively respond to the needs by investigating patients’ requests and improving caregivers’ skills in a caring environment. In CNT, however, the principal issue is the risk of injury to the subjects during training due to a trainee’s ineptitude. Therefore, it is necessary to train experts who can competently manage various situations and meet the needs of individuals with diseases (Kitajima et al., 2014) according to medical and healthcare systems’ advances.

Two of the critical challenges in using PRCNTs in daily life are patient transfer and rehabilitation. For patient transfer, caregivers commonly perform tasks in hospitals, vehicles, and homes to move patients with mobility problems or those who need a wheelchair (Huang et al., 2015). The complicated tasks in patient transfer include parking a wheelchair, mutual hugging, standing up, pivot turning, and sitting down in a wheelchair. Huang et al. (Huang et al., 2015; Huang et al., 2017; Huang et al., 2016) proposed the patient robot for transfer and investigated the effect of practice on skill training through robot patients. In addition, Lin et al. (2021) proposed a PRCNT to assist patients in sit-to-stand postures. Many studies involving the use of patient robots in training systems for daily life activities have yielded notable outcomes.

In the case of rehabilitation, patients with musculoskeletal disorders may experience limited muscle and joint movement due to various symptoms (stiffness, contraction, or weakness of muscles). Thus, caregivers or therapists must periodically ask patients to undergo rehabilitation. Because novices may apply unnecessary force to the joints when performing rehabilitation or stretching for patients, caregivers must practice sufficiently in advance to not stress the joints or skin of a patient with musculoskeletal disorders when providing care or treatment. Mouri et al. (2007) developed a robot hand that evaluates the joint torque with a disability for rehabilitation training. Fujisawa et al. (2007) proposed an upper-limb patient simulator for practical experience training. Their simulator can simulate elbow joint stiffness, allowing trainees to improve their skills in stretching during physical therapy. Although studies have indicated that patient simulator robots are gaining increasing attention, simulators for CNT remain insufficient. Simulated robots have been developed in many studies; however, a human-robot interaction system in which simulated robots can directly interact with humans is yet to be developed, as shown in Table 1. In addition, the simulated robot for CNT still relies on post-evaluation using statistical analysis, and a more advanced feedback method is required for the interaction between users and robots.

**TABLE 1:** Comparison between the related work and the present study in this work.

References	Description	Feedback method
Matsumoto et al. (2018)	Whole-body robotic simulator of an elderly person for evaluating robotics devices for nursing care	Not provided
Fujisawa et al. (2007)	Upper limb simulator (elbow joint) of a patient for evaluating robotics devices for nursing care	Not provided
Huang et al. (2015), Huang et al. (2017), Huang et al. (2016), Huang et al. (2014)	Whole-body robotic simulator of a patient for transfer training	Voice-based feedback
Wang et al. (2012), Wang et al. (2013)	Upper limb simulator of a real human for neurologic examination training	Not provided
Ishihara et al. (2011)	Child robot for understating the caregiver-child attachment relationship	Facial expression-based feedback
Present study	Patient robot with robotic pain inference model for rehabilitation training	Facial expression-based feedback

To achieve an effective CNT feedback system, it is important to design patient robots for CNT that can express feelings of pain states like humans through visual feedback. Robust feedback methods that robots can use to provide feedback to learners can be based on visual information and sound. Huang et al. (Huang et al., 2015; Huang et al., 2017; Huang et al., 2016; Huang et al., 2014) proposed the patient robot for transfer and investigate the effect of practice on training skills though robot patient with voice-based feedback. However, the visual feedback is the most effective method in terms of practice for caregivers because they need to periodically investigate whether the patient is feeling pain or not. In particular, it is imperative to observe painful expressions on the patient’s face because the patient may experience a burden in communicating with caregivers. Pain is an immediate response that protects the human body from tissue damage and can be observed as a subjective measure. When humans are subjected to physical pressure from external factors, most humans usually express pain through facial expressions, voice, and physical responses. In 2011, Ishihara et al. (2011). presented the realistic child robot Affetto, which aims to improve understanding and interaction between the child and caregiver to support the child’s development. Affetto can sense a touch or hit by detecting changes in pressure from synthetic skin. Based on this pressure sensation, Affetto is being developed as a robot capable of expressing pain and emotions with a painful nervous system. Thus, by applying the pain response system to a robotic system, it is possible to build a robotic system that can feel pain as a real human does when subjected to physical pressure from external factors.

**FIGURE 1:**
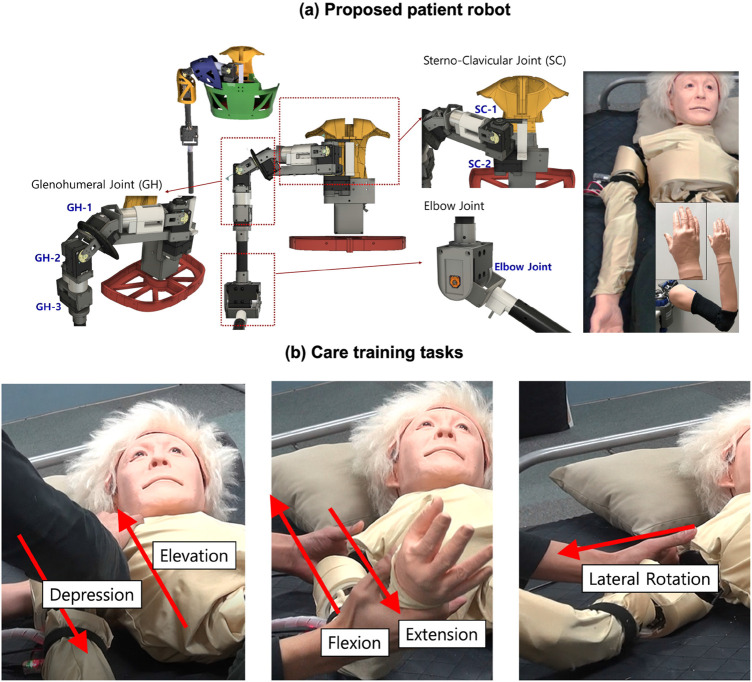
Proposed patient robot in previous studies (Lee et al., 2019; Lee et al., 2020) **(A)** proposed patient robot **(B)** care training tasks using the robot.

Based on the aforementioned issues and motivation, we consider the patient robot as a care training assistant to simulate a patient with specific musculoskeletal symptoms as shown in Figure 1. Furthermore, as previously stated, an advanced feedback system for care training is a significant issue for the proposed care training system. Therefore, this study presents a patient robot that can express feelings of pain states like humans and examines a visual feedback method that allows the user to respond immediately to the robot’s pain state during care training. The objectives of the method for pain inference and expression of the patient robot introduced in this work to achieve the goals are as follows:

• To provide automated quantitative assessment feedback on care training to the caregiver

• To develop a method for pain inference for the care training system

• To build a database to generate a robot’s avatar by recruiting subjects of various ages

• To express the current pain state through the robot’s avatar

## 2 RU-PITENS database

Most care training studies use statistical or empirical techniques to manually analyze the results. These methods are suitable for the analysis of each parameter and are easy to use when investigating the effects of parameters on care training. However, it is difficult for trainees to evaluate their treatment quantitatively in a real-time system, and there is a limitation in terms of automatically calculating the final score after finishing care education. Therefore, there is a need to present a method for automatically inferring the care and nursing skills as well as the robot’s pain level, based on data acquired from sensors mounted on the robot. In addition, caregivers should periodically investigate whether the patient is feeling pain and observe painful expressions on the patient’s face during care because the patient may have difficulty communicating with caregivers. This section describes robotic pain expression based on the pain inference results. In this study, the novelty is that it presents the avatar-based feedback system to express the pain of a patient robot to improve caregivers’ skills in a training environment.

### 2.1 Participants

Forty-one Japanese subjects (26 men and 15 women) participated in this experiment (Table 2). Pain expression facial images from 41 subjects, including 26 men (mean age 46.6 ±3.4 ) and 15 women (mean age 55.8 ±2.4 ), were acquired for pain image analysis. None of the subjects had a history of neuropathy or pain symptoms, facial muscle disorders, trauma, or medication in past. Further, each subject was briefed on the study’s purpose, and all participants have agreed to experience by signing a consent form. Researchers observed to ensure their safety during the experiment. In addition, consent was sought to use the database containing facial images as an openly available database, and all participants agreed. This study was approved by the Institutional Review Board (IRB) of Ritsumeikan University (BKC-2019-060).

**TABLE 2:** Demographics of the participant’s gender and age in pain intensity using the TENS device (RU-PITENS) database.

Pain intensity using the TENS device (ru-PITENS) database						
Age range						
**Gender**	**Measure**	**20–29**	**40–49**	**50–59**	**60–69**	**Total**
Male	N.S	11	5	5	5	26
Female		—	5	5	5	15
Total		11	10	10	10	41
Male	M.A	23.7	45.0	53.6	64.0	46.6
		(2.1)	(4.6)	(3.0)	(3.9)	(3.4)
Female		-	45.2	56.0	66.2	55.8
			(2.3)	(1.6)	(3.3)	(2.4)
Total		23.7	45.0	53.6	64.0	47.2
		(2.1)	(4.6)	(3.0)	(3.9)	(2.9)

Note: N.S and M.A indicate the number of subjects and the mean age, respectively. Numbers in parentheses are standard deviations.

### 2.2 Data acquisition

Transcutaneous electrical nerve stimulation (TENS) has been used to stimulate muscles to evaluate the pain state (Jiang et al., 2019), (Haque et al., 2018) because the TENS is inexpensive, non-invasive, and easy to utilize compared to other devices (thermal or pressure stimulation). TENS devices are frequently used for muscle therapy in daily life and can induce acute painful situations with high levels (frequency).

**FIGURE 2:**
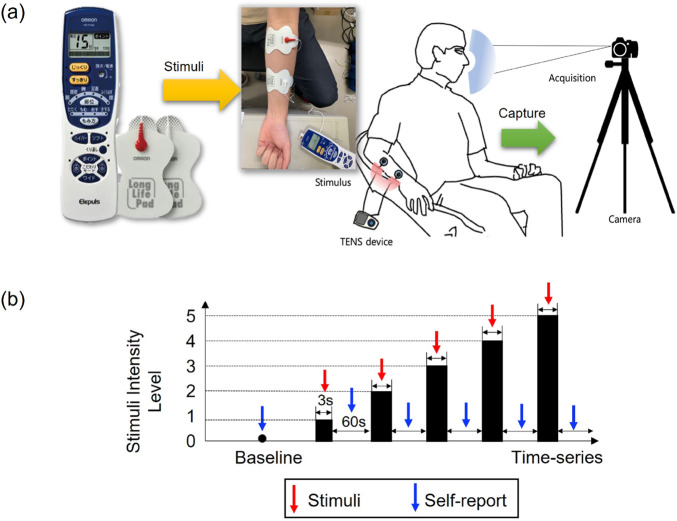
Environment of pain facial expression images in RU-PITENS database **(A)** environment **(B)** protocol.

To collect a painful database in this study, we used the *HV-F128* electric therapy device (OMRON Co., Ltd., Japan). Two *HV-LLPAD* durable adhesive pads (OMRON Co., Ltd., Japan). The experiment was performed until the subjects could no longer tolerate the pain when the TENS level (from one to five) was increased or reached the maximum level (the output had five levels and its frequency range from 0 to 1,200 Hz). Figure 2 shows the environment of the pain stimulation using a TENS device and the acquisition of a pain expression image. The participants sat on a chair, attached a TENS to their right arm, and stared at the front camera during the experiment (Figure 2A). The arm muscles were stimulated for approximately one to 3 seconds through the TENS device, and face images were acquired from the camera at three to five frames per second. Figure 2B illustrates the protocol for the experiment, and the subjects performed the self-assessment manikin (SAM) (Lang et al., 1997) scales for arousal (SAM-A), SAM scales for valence (SAM-V), and subjective pain level assessment at the end of each level.

**FIGURE 3:**
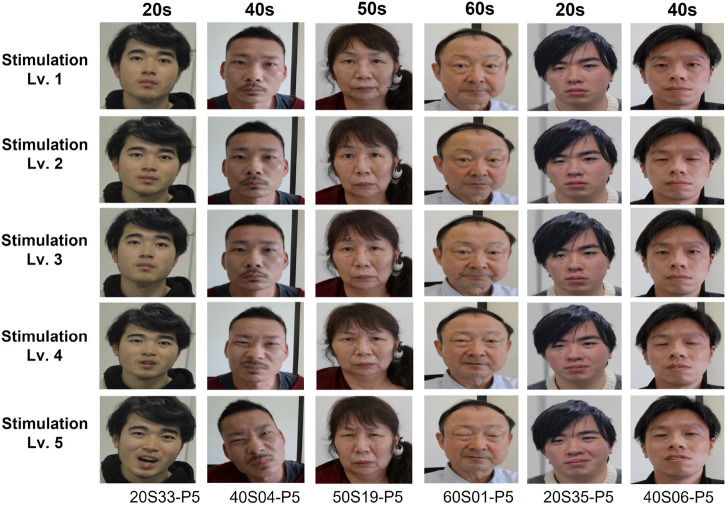
Example of the pain images in RU-PITENS database.

A total of 13,773 frames of images were acquired from all subjects. Figure 3 illustrates an example of the acquired pain facial expression images. The experiment for building this database was conducted at the Ritsumeikan University in Japan, and it was released as an open database: https://github.com/ais-lab/RU-PITENS-database. Further information is provided in the following subsections.

## 3 Pain intensity and expression

### 3.1 Network-based pain intensity

The optimal objective of this study is to create an avatar representing pain facial expressions from sequential pain images in the RU-PITENS database. Although the proposed method generates an avatar using the face image in the RU-PITENS database, the pain expression images in this database do not have a quantitative value (reference) for the expression intensity from the onset to the cessation of pain state. Therefore, it is necessary to measure the pain intensity using a verified model. In this study, the *Siamese* and *Triplet* networks were used to measure the intensity of pain from pain images for the following reasons:

• Because this study aimed to develop the avatar-based expression system based on sequential pain intensity estimation, models that could measure the change in pain intensity between the previous image and the current image were required.

• It is difficult to distinguish the type of pain and to provide an accurate pain label to new input data because pain is subjective information that can be measured differently depending on the individual.

According to the aforementioned considerations regarding the use of the Siamese network, the pain intensity from pain images in the RU-PITENS database was measured using the *Siamese* and *Triplet* networks. This network (Bromley et al., 1994) provides one output, which indicates the similarity between two inputs. In many studies (Hayale et al., 2019; Liu et al., 2020; Sabri and Kurita, 2018), it has been used as a system for analyzing facial expressions because facial expressions gradually change while expressing an emotion from the current emotion to the next emotion. The Siamese or Triplet network has two or three sister networks (sub-networks) with the shared weight and structure, which consists of a layer for computing the distance of the feature vectors from the sister networks. For loss learning, we adopted the exponential loss (Sabri and Kurita, 2018) using $Loss_{\mathrm{siamese}}$ (Equation 1) and $Loss_{\mathrm{triplet}}$ (Equation 2).

$$Loss_{\mathrm{siamese}}=\min\underset{x_{0},x_{1}}{\sum }\exp\left(g_{0}\left(x_{0}\right)-g_{1}\left(x_{1}\right)\right)$$ (1)

$$\begin{array}{ll} Loss_{\mathrm{triplet}} & =\min\underset{x_{0},x_{1},x_{2}}{\sum }\exp\left(g_{0}\left(x_{0}\right)-g_{1}\left(x_{1}\right)\right)-\exp\left(g_{0}\left(x_{0}\right)-g_{2}\left(x_{2}\right)\right)) \end{array}$$ (2)

where $g_{i}\left(x_{i}\right)$ indicates each branch output. In the loss of the Triplet network, $g_{0}\left(x_{0}\right)$ is the anchor input, the distance from the anchor to the positive input is minimized, and the distance from the anchor to the negative input is maximized.

**FIGURE 4:**
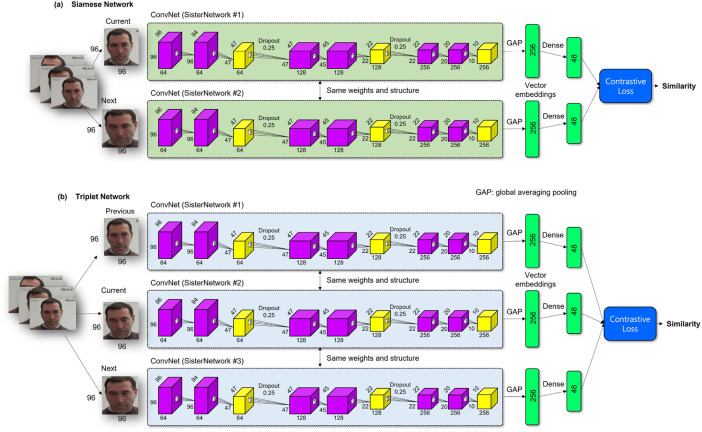
Structure of the Siamese network for pain intensity evaluation in the RU-PINTENS database **(A)** Siamese Network **(B)** Triplet Network.

As shown in Figure 4, the sharing networks have basic ConvNet model’s structures, and the network architecture contains three ConvNet layers and a fully connected layer with 48 units based on the results of the hyper-parameters that have been empirically tuned (Lee et al., 2024). The fully connected layer that calculates the output of the two sister networks is added to the last layer for connecting the shared networks.

### 3.2 Avatar-based pain expression

Based on the pain intensity of pain images determined using the Siamese and Triplet networks, we obtained the quantitative level for pain images in the RU-PITENS database, and the sequential pain expression can be expressed through the avatar. The robotic head provides natural facial expressions that can sustain the interaction between a robot and an individual. Berns and Hirth (2006) proposed a method for controlling robotic facial expressions using the robot head *ROMAN*. Kitagawa et al. (2009) proposed a human-like patient robot to improve the ability of nursing students to inject a vein in the patient’s arm; the robot was designed with the aim of being operated to express various emotions such as neutral, smile, pain, and anger. Although the robot’s expression can be communicated in various manners, using a projector has the advantages of low expense and comfort. One of the most significant advantages is that the facial features (age, gender, specific person, etc.) can be easily and conveniently transformed. The visual feedback that may be obtained using a projector can represent various realistic facial expressions. Maejima et al. (2012) proposed a retro-projected 3D face system for a human-robot interface. Kuratate et al*.* (Kuratate et al., 2013; Kuratate et al., 2011) developed a life-size talking head system (Mask-bot) using a portable projector. Pierce et al. (2012) improved the preliminary *Mask-bot* (Kuratate et al., 2013; Kuratate et al., 2011) by developing a robotic head with a 3-DOF neck to study human-robot interactions. According to the study of (Pierce et al., 2012), The meaningful advantage of the robot’s avatar using a projector is that it may not depend on complicated mechanical systems such as motors. Therefore, many motors do not need to be handled to change the facial expression, and it is easy to modify the avatar or the robot’s head form. Hence, a projector-based robotic head that expresses the pain and emotions for care training is proposed in this study.

**FIGURE 5:**
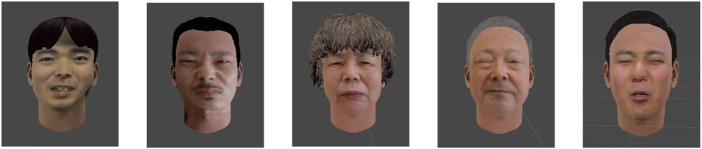
Example of avatar generation using the facial images in RU-PITENS database.

To create an avatar object (.obj), a commercial avatar SDK (Itseez3D, Inc., CA, USA) was used in this study. The patient robot’s avatar, which can express pain, was converted from the original image (.jpg) to an avatar object (.obj) through the avatar SDK added to the Unity program (Unity Technologies, Inc., CA, United States). Figure 5 illustrates the robot’s avatar from the participant’s facial image from the RU-PITENS database. The avatars generated according to the frames of all the original images were classified into five pain groups (PGs): PG1, no pain at all; PG2, weak; PG3, moderate; PG4, strong; and PG5, very strong. In other words, five types of pain avatars can be expressed based on the pain level of the robot that feels pain during CNT.

**FIGURE 6:**
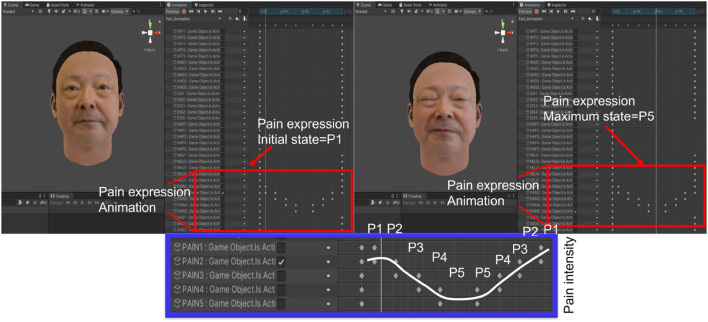
The method to animate the avatar’s facial expressions.

To generate the animation, Unity’s animator was adopted to animate the avatar’s painful facial expressions. Each group maintained an interval of approximately 0.5 s, and animation according to the facial expressions of the avatars was completed, as shown in Figure 6A. Figure 6B depicts the expression transition of the avatar as it changes from neutral to a specific expression (painful expression in this study) and then returns to the neutral state.

## 4 Results and discussions

### 4.1 Results of the questionnaire in RU-PITENS database

**FIGURE 7:**
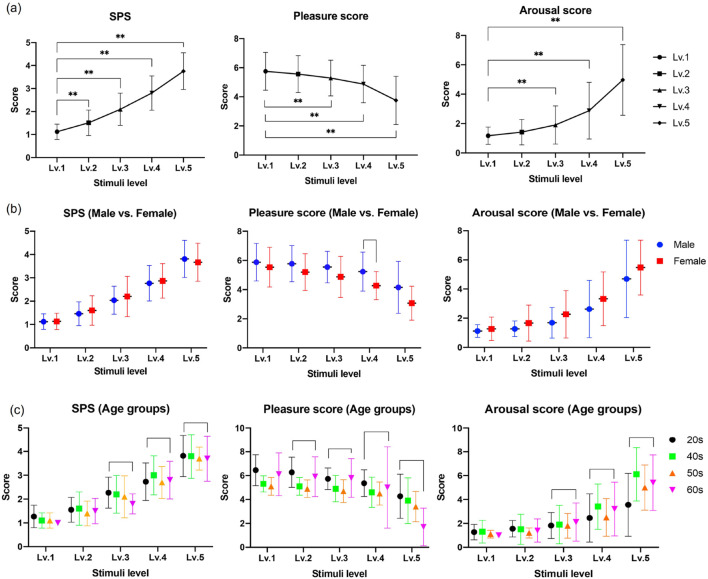
Result of the survey in RU-PITENS database **(A)** All subjects **(B)** Survey results according to gender group **(C)** Survey results according to age groups (20 s, 40, 50 s, and 60 s). The test methods were used the analysis of variance (ANOVA) and Tukey’s method (*post hoc*analysis). The significant level was set at α = 0.05. An asterisk (*) indicates statistical significance at p< 0.05, and double asterisk (^**^) indicate statistical significance at p< 0.01. SPS and Lv indicate the subjective pain score and the stimuli level.

As shown in Figure 7, to examine the result of pain intensity in our RU-PITENS database, subjects responded to the questionnaire instantly after the stimulation tests for each level. The survey consists of a visual analog scale (VAS) and a subjective pain score (SPS). The VAS is easy to use and is frequently used to assess variations in the intensity of pain. In this experiment, the SPS survey was designed as a subjective indicator of pain. In terms of value, SPS can be classified as follows: no pain at all = 0, very faint pain (just noticeable) = 1, weak pain = 2, moderate pain = 3, strong pain = 4, and very strong pain = 5. As shown in Figure 5A, the SPS continuously increased according to the stimuli levels, and there were statistically significant differences among stimuli levels (F = 164, p < 0.01, ANOVA test). For the pleasure score, the score decreased with respect to the stimuli level, indicating that the pain stimulus had a negative effect on the subject’s emotions. There was a difference of approximately 2.0 between the maximum level (Lv.5) and the minimum level (Lv.1) in SPS (Q = 9.89, p < 0.01, Tukey’s *post hoc* test). An increase in the arousal score with the stimuli level suggested a negative effect of the stimulus and showed a difference of approximately 3.81 between the minimum (Lv.1) and maximum (Lv.5) stimulation levels (Q = 14.42, p < 0.01, Tukey’s *post hoc* test). Additionally, according to the evaluation of all parameters for gender, there was no statistically significant difference between the men and women groups. However, when the survey statistics were analyzed by age group (20 s, 40 s, 50 s, and 60 s), there were differences in each survey result. In the case of SPS and arousal scores, there was a statistically significant difference in all age groups from Lv. to Lv.5 (ANOVA test), and there was a significant difference between Lv.2 and Lv.5 (ANOVA test) in the pleasure score. Based on these results, the pain images in Lv.5 (maximum stimulation level) were utilized to generate the robot’s facial avatar.

### 4.2 Results of siamese and triplet network-based pain intensity

To train the Siamese and Triplet networks, the UNBC-McMaster database was used to train a model to measure pain intensity from facial images in the RU-PITENS database, which was used to generate pain expression avatars. The UNBC-McMaster shoulder pain database (Lucey et al., 2011) contains pain images from 25 patients with shoulder pain collected through an experiment on shoulder range of motion. The UNBC-McMaster database can be used for model training because it contains the *Prkachin and Solomon Pain Intensity* (PSPI) score, which is the ground truth of pain level. The PSPI (range from 0 to 15) is a score that measures the level of pain in facial expressions, which was first proposed in (Prkachin, 1992), and is calculated by several action units (AUs) using a facial action coding system (FACS) (Ekman et al., 2002). AUs are the visible indicators of the operation of facial muscles. The PSPI score can be calculated as the sum of several AUs including AU4 (brow lower), AU6 (cheek raiser), AU7 (eyelid tightener), AU9 (nose wrinkle), AU10 (upper lip raiser), and AU43 (eyes closed). The PSPI value was the basic factor in evaluating the model generated to calculate pain intensity from pain images.

**FIGURE 8:**
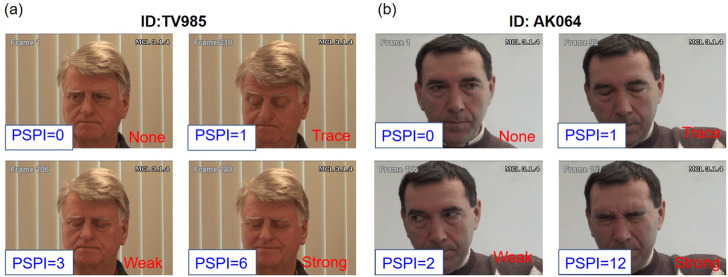
The method to animate the avatar’s facial expressions **(A)** animator **(B)** expression transition.

Before training the model, the UNBC-McMaster database must balance the number of data entries in each class because the data are unbalanced and skewed. Based on the PSPI score (ranging from 0 to 15), the pain images in the UNBC-McMaster database can be divided into four pain labels: none (PSPI = 0), trace (PSPI = 1), weak (PSPI = 2 and 3), and strong (PSPI >= 4). As shown in Table 3, the total number of data entries from UNBC-McMaster is 48,398. Because pain is subjective and there are no clear criteria for classification, many studies arbitrarily classify the PSPI labels. Therefore, in this study, the PSPI label was decided according to the standards proposed in the study of (Bargshady et al., 2020) by considering the unbalanced data in the UNBC-McMaster database. This study included only 1,730 images in each pain group for balanced data based on a minority grade (pain label: strong (PSPI >= 4)), and the data were extracted randomly. To use this database for research purposes, an end user license agreement was submitted to the Affect Analysis Group at Pittsburgh (Lucey et al., 2011). Figure 8 illustrates an example of pain images in the UNBC-McMaster shoulder pain database.

**TABLE 3:** Definition of pain states based on PSPI for classifying classes in UNBC-McMaster shoulder pain database (Lucey et al., 2011).

Prkachin and solomon pain intensity (PSPI)	Pain state	Number of images
0	None	40,149
1	Trace	3,037
2 and 3	Weak	3,482
from 4 to 15	Strong	1,730

Note: PSPI indicates Prkachin and Solomon Pain Intensity score.

Table 4 shows the confusion matrix for the user’s facial expression. For testing the models, input images of × resolution were input to the networks to train the Siamese and Triplet networks (learning rate = 0.1, batch size = 64, and epochs = 30; these hyper-parameters were adjusted empirically to determine the best performance.). A total of 2,856 pairs of samples were used for the training set, and the remaining samples were used for the testing set in the UNBC-McMaster database (Lucey et al., 2011). For the network’s estimation, we calculated the Pearson correlation coefficient (PCC) and the mean absolute error (MAE) to estimate the sequential pain intensity using the networks compared to the ground truth (PSPI). The PCC is a statistical test that calculates the relationship between two variables. It has a value between −1 and 1, and the nearer it is to 1 (positive correlation) or −1 (negative correlation), the higher the correlation. 4 shows that the Siamese network with the three-layer model had the optimal estimations (PCC = 0.87 and MAE = 3.13).

**TABLE 4:** Confusion matrix for the user’s facial expression.

	Siamese network	PCC	MAE
Siamesenetwork	ConvNet-Layer 3	0.87	3.13
	ConvNet-Layer 4	0.79	4.89
	ConvNet-Layer 5	0.55	6.11
Triplet network	ConvNet-Layer 3	0.86	3.39
	ConvNet-Layer 4	0.76	4.31
	ConvNet-Layer 5	0.49	7.59

**FIGURE 9:**
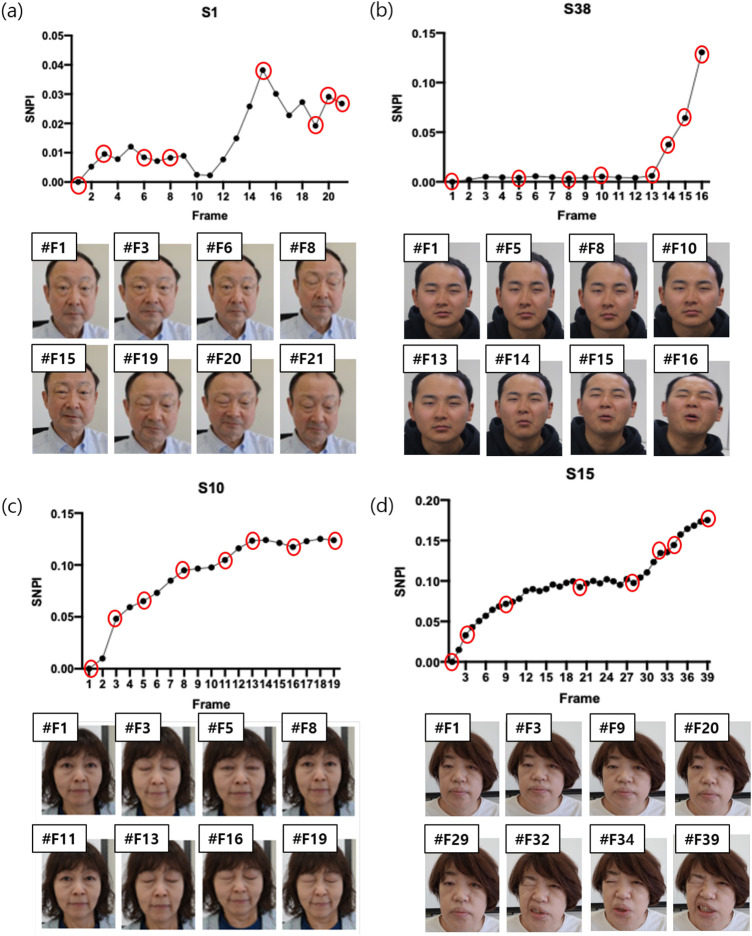
The result of pain intensity using Siamese network (SNPI) from subjects in RU-PITENS database **(A)** S1 **(B)** S38 **(C)** S10 **(D)** S15.

The results of most subjects showed that the intensity of facial pain increased with the intensity of the electrical stimulation, as shown in Figure 9. Comparing the pain intensity extracted from the facial image (SNPI) and the questionnaire, considering the results of S1, S10, S15, and S38, the four participant’s SPS, pleasure, and arousal scales averaged approximately 4.0 (out of 5 points), 3.0 (out of 9 points, the higher the score, the more positive) and 5.75 (out of 9 points), respectively. Thus, in general, as the stimulation intensity increased using the TENS device, the intensity of pain obtained from the facial expression and the questionnaire results showed a similar pattern. Consequently, it can be concluded that facial images, including pain intensity, can be acquired through stimulation using a TENS device.

### 4.3 Results of the avatar-based pain expression

**FIGURE 10:**
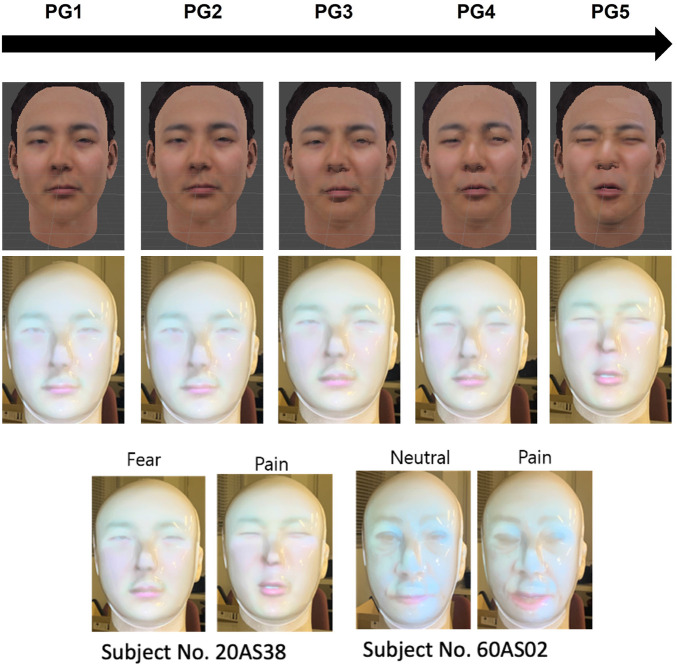
Avatar-based robot’s pain expression.

Figure 10 illustrates an example of the avatar for pain expression. This study attempted to create an avatar of a patient robot that considered various age groups and genders without depending on a specific target’s facial shape and appearance. Several images for avatars were used based on the RU-PITENS database. The pain images in the RU-PITENS database are images captured when the subjects felt pain and are divided into five pain groups according to the intensity of pain obtained from the image.

A projector was used in the experiment to express the robotic facial expressions. Figure 10 depicts the testing of the projector-based robotic head for emotion and pain expression. The projector is placed in front of a translucent facial mask, and the avatar’s expression is represented based on the facts obtained from the patient robot or the user. Moreover, the command is transmitted to the Unity program on the personal computer.

**FIGURE 11:**
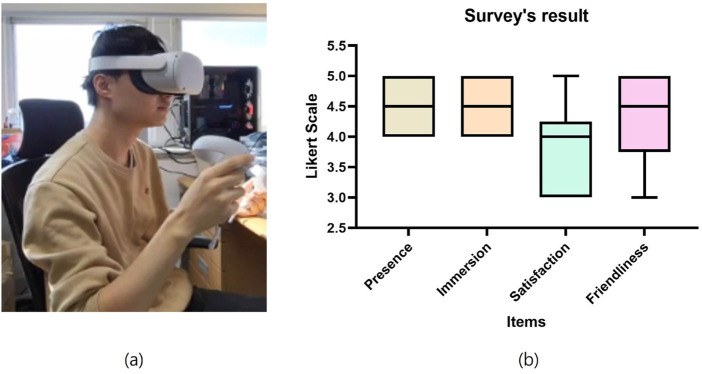
Result of subject’s survey for avatar **(A)** experimental environment **(B)** survey result.

For the quantitative evaluation of the generated avatars, we used a survey to assess the users’ satisfaction, as shown in Figure 11. The subjects who participated in the survey tried to use the avatar’s interaction in a VR environment. The survey’s items consist of four types: presence, immersion, satisfaction, and friendliness. Additionally, subjects can estimate the avatar’s interaction through the Likert scale (0–5) for each survey item. As shown in Figure 11, most participants were satisfied with all items, and in particular, the average score for presence and immersion was high at 4.50.

### 4.4 Limitations

The integration system for practical care training based on visual feedback was proposed to improve the care skills of caregivers. The most crucial advantage of a projector-based robot head is that it is easier and more convenient to change the avatar than mechanical or physical methods. The avatar with various age groups and genders can be expressed. Therefore, our proposed system provides an environment for learners to train how the patient’s mood changes and respond to the patient’s pain according to the patient’s personality and pain sensitivity by applying the patient’s face picture and personality to the patient robot in advance in the future. Despite the advantages described above, the proposed system have obvious limitation. Our study has not yet proven the effectiveness of the proposed system in a CNT environment and its impact on learning outcomes. Addressing these limitations will significantly enhance the contributions and impact of their work in the field of CNT and human-robot interaction.

## 5 Conclusion

In this study, we focused on technological improvements in advanced care and nursing training systems by developing patient robots based on pain expression. Our contributions can be summarized as follows:

• Typically, the user relies on the parameter’s result or graph of the robot’s sensor data for performing care training. Because the user’s gaze tends to be concentrated on the robot’s joints and joint movements, it is difficult to determine how the caregivers conduct their care tasks satisfactorily. However, this study’s proposed visual feedback approach allows the caregivers to receive feedback from painful facial expressions in the robot and receive more attributes via advanced human-robot interaction.

• We addressed the database of the facial images with pain expressions from 41 Japanese people to generate a robot’s pain avatar and the database will be disclosed as an open database to expand the scalability of the research related to the pain expression in various fields.

Consequently, it is anticipated that these visual indicators can play a crucial role in achieving the purpose of effective care education that allows users to react immediately.

## Data Availability

The datasets presented in this study can be found in online repositories. The names of the repository/repositories and accession number(s) can be found in the article/supplementary material.
